# Changes in coronal alignment of the hip joint after medial opening wedge high tibial osteotomy

**DOI:** 10.1007/s00590-022-03269-0

**Published:** 2022-04-25

**Authors:** Jaison Patel, Reece Patel, Joel Melton

**Affiliations:** 1 Department of Trauma and Orthopaedics, Addenbrookes Major Trauma Unit, Cambridge University Hospitals, Cambridge, UK; 2 School of Clinical Medicine, University Of Cambridge, Cambridge, UK

**Keywords:** High tibial osteotomy, Knee, Osteoarthritis, Hip biomechanics, Tomofix, Varus

## Abstract

**Purpose:**

An observation was made by the senior author of this paper that patients reported changes in their hip function after a medial opening wedge high tibial osteotomy (MOHTO) for varus pattern osteoarthritis. Alignment changes at the hip after MOHTO have not been previously documented. This study assesses coronal alignment changes at the hip after MOHTO.

**Methods:**

We retrospectively analysed pre- and post-operative lower limb alignment radiographs of patients who underwent MOHTO. The medial proximal tibial angle (MPTA) and mechanical axis deviation (MAD) were measured to assess the alignment changes created by the MOHTO. The coronal alignment changes at the hip were evaluated using the mechanical greater trochanter angle (MGTA).

**Results:**

29 osteotomies in 27 patients were included in this study. Results showed MOHTO created alignment changes at the hip. A positive correlation was found between the size of the correction at the knee and the subsequent changes at the hip. The change in the MGTA had a stronger correlation with the MAD than with the change in MPTA (*r* = 0.684 vs. 0.585). It was found that age, weight, height and BMI had no significant influence on these correlations.

**Conclusions:**

Increased correction by the MOHTO lead to increased change in the coronal alignment of the hip. These changes are likely to result in an alteration in the weight bearing portion of the femoral head and the function of the abductors and we recommend assessing the hip joint as part of pre-operative planning.

**Level of evidence:**

Prognostic level IV.

**Supplementary Information:**

The online version contains supplementary material available at 10.1007/s00590-022-03269-0.

## Introduction

High tibial osteotomy (HTO) is a commonly used procedure to treat medial compartment osteoarthritis of the knee. The medial opening wedge high tibial osteotomy (MOHTO) is considered the work horse osteotomy of the knee when managing medial compartment osteoarthritis [1]. In a patient with genu varum, the mechanical axis causes increased forces to be projected through the medial compartment of the knee, which subsequently promotes a degenerative process [2, 3]. A MOHTO laterally translates the mechanical axis to offload the medial compartment and load the lateral compartment. Fujisawa has previously described the “optimum correction” to be when the Mikulicz line crosses 62% across the tibial plateau medially to laterally [3]. There is however little biomechanical reasoning behind this figure and even when the weight-bearing line crosses the midpoint of the knee 70% of the load is already through the lateral compartment [4].

The biomechanical relationship between the hip, knee and ankle is important. The three joints work as a kinetic chain whilst walking [5] and so alignment changes of the knee may affect alignment changes of the ankle and hip. As surgeons, it is crucial to be able to predict the impact of your surgery on the limb as a functional unit.

An observation was made by the senior author of this paper that patients in this group reported a change in their hip function after MOHTO. Observations of the hip in patients with knee malalignment have been made previously [5]. The current literature on the hip after MOHTO is limited to case reports [6]. Changes in the coronal alignment of the ankle after MOHTO have been studied in the past [7–10]. There is a paucity of existing literature describing the effects of a MOHTO on the hip joint. The primary aim of this study is to assess whether coronal alignment changes at the hip occur after MOHTO and if these changes correlate with the degree of correction created. The secondary aim of this study is to assess whether these changes, if present, are affected by patient demographics such as age, weight, height, and BMI.

## Materials and methods

Approval for this study was granted by the local institute. All patients that underwent MOHTO at a large tertiary centre from 2015 to 2019 were retrospectively identified. Surgery was performed by a single experienced fellowship-trained knee surgeon. Indications for surgery included isolated medial compartment osteoarthritis with a genu varum deformity of the proximal tibia, absence of inflammatory arthropathy, no fixed flexion deformity, and non-smokers.

**Fig. 1:**
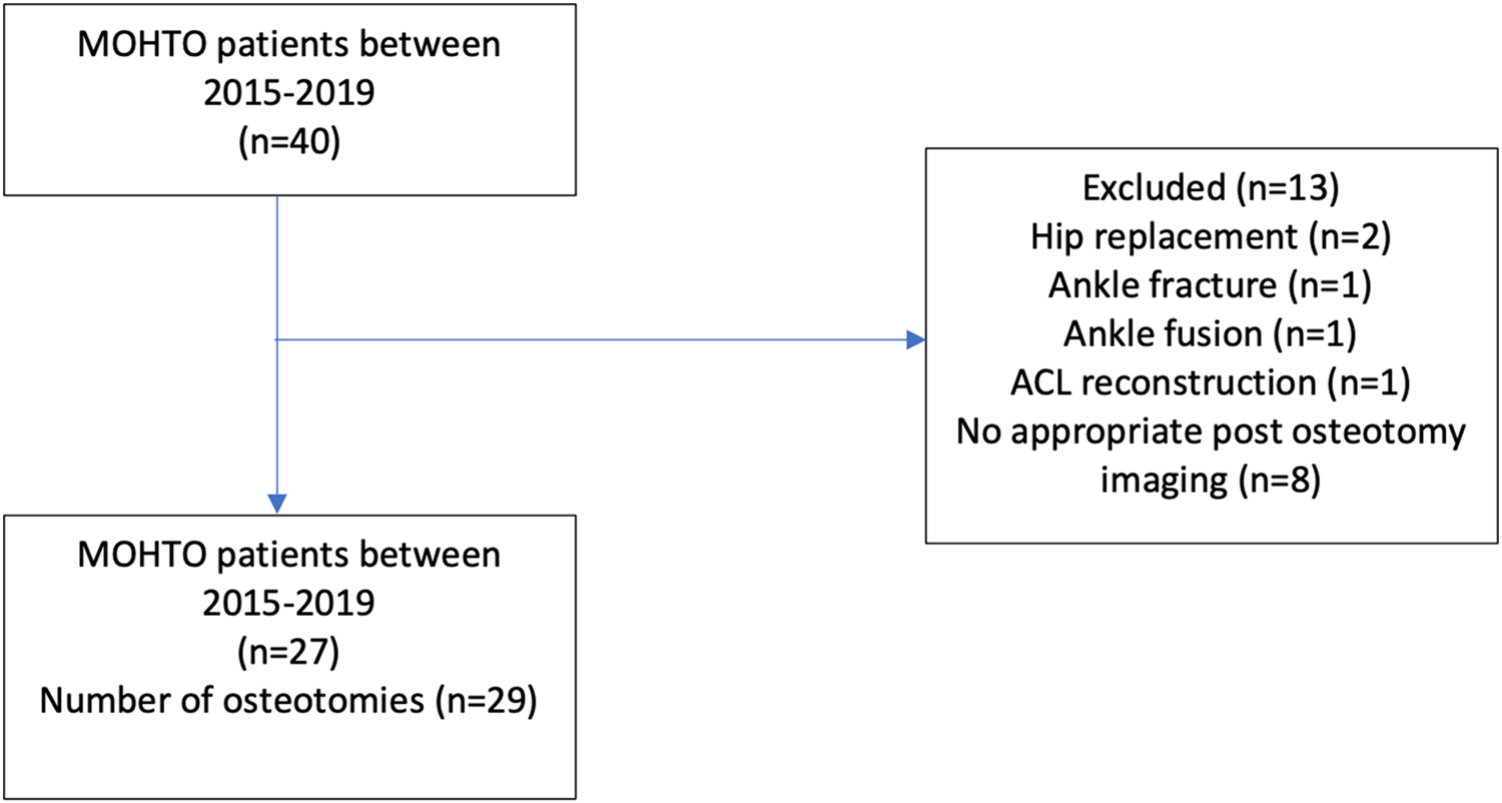
Demonstrating the included osteotomies in this study and the criterion for excluding osteotomies

Inclusion criteria included medial compartment osteoarthritis as the indication for surgery, single operating surgeon and Tomofix plate fixation. Exclusion criteria included: other surgery of the ipsilateral limb, combined procedures such as double osteotomies or ligament reconstruction, severe patellofemoral disease and lateral compartment osteoarthritis. Patients were identified through an online healthcare records database (EPIC systems, United States). A Total of 40 osteotomy procedures were identified through the online database. After exclusion criteria were applied a total of 29 osteotomies in 27 patients were included in the study (Fig. 1). Follow-up imaging showed the mean time between the date of surgery and last x-ray ranged from 3–50 months (M = 17.32, SD = 12.26).

Full length, weight-bearing radiographs were obtained of the lower limbs bilaterally. All imaging was carried out in a single centre with standardised protocols. The focal film distance was standardised at 180 cm with a stick ruler placed behind the patient. Patients stood on a box to ensure ankle joints were included in the imaging with both patellae facing forward to obtain true AP projections of the knee. Patients were asked to keep knees fully extended. Three images were digitally stitched to produce one image.

A minimally invasive medial approach was used for all patients. A temporary wire was used to guide the direction of the osteotomy. Biplanar osteotomies were created with an angle of 100–120 degrees in the coronal plane proximal to the patella tendon insertion due to its various documented advantages [11]. All patients underwent a MOHTO aiming for a correction to promote lateral compartment loading. Osteotomies were held using a TOMOFIX plate (DePuy Synthes, Switzerland).

Analysis of the images was carried out by a senior orthopaedic registrar with an interest in knee surgery. Standardised reference points were created for each analysis using:

- The reference point for the femoral head was created by drawing the best fit circle around the femoral head that crossed 3 points circumferentially at the 12, 3/9 and 7/5 o’clock positions depending on laterality.
- The centre of the knee was taken as the midpoint at the base between the tibial spines.
- The centre of the ankle joint was taken as the mid-point of the talus.

### Assessment of the correction

**Fig. 2:**
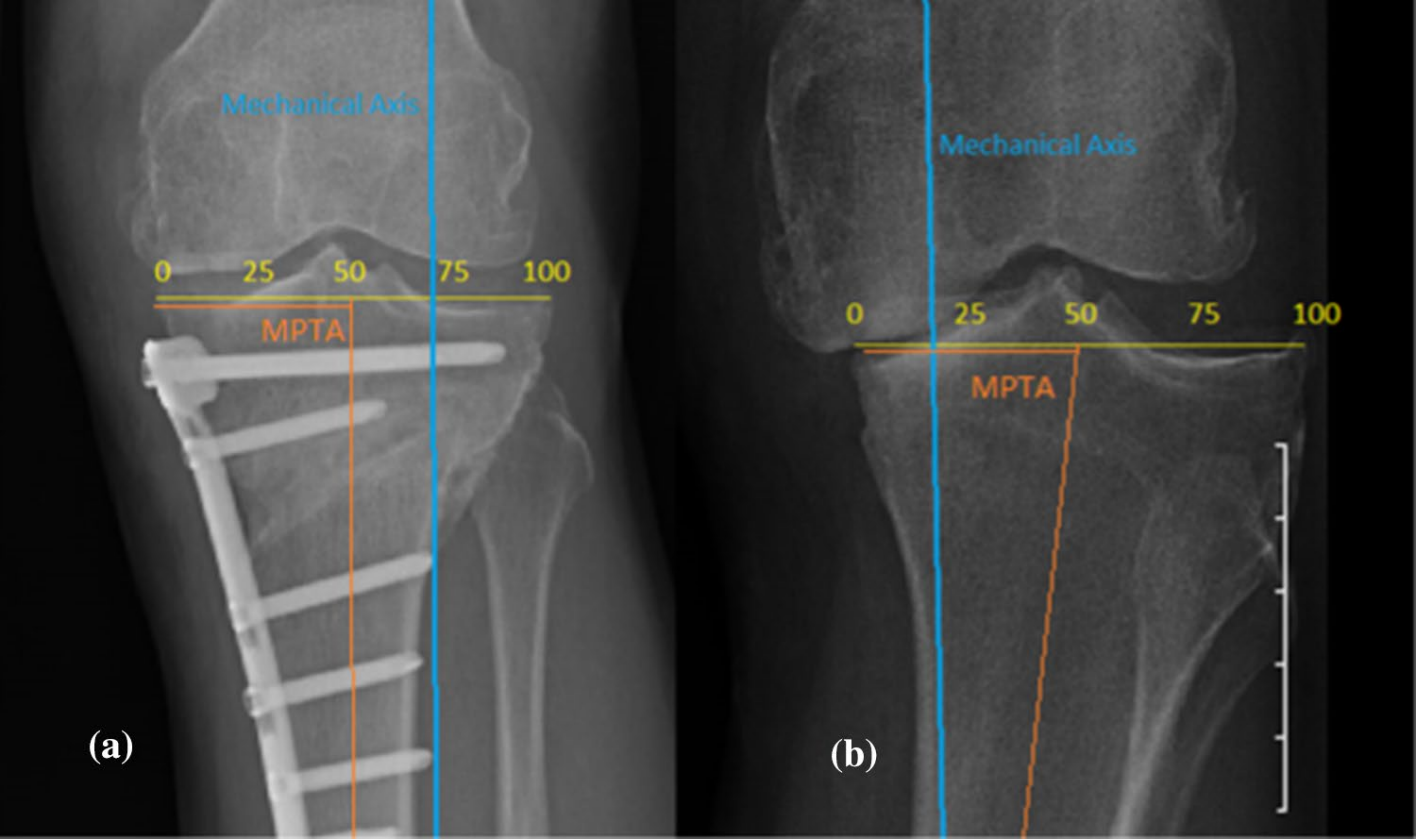
The MPTA and MAD post (**a**) and pre (**b**) operatively. In image (**a**) the MAD crosses 73.5 and in (**b**) 19.6 giving an overall change of 53.9. The MPTA in figure **a** is 92.4 degrees and 78.6 degrees in **b** giving an overall change of 13.8 degrees. These were calculated for all osteotomies to quantify the changes created by the osteotomy

Two methods were used to quantify the changes created from the MOHTO. These were calculated pre- and post-operatively to calculate the change (Fig. 2).

1. The medial proximal tibial angle (MPTA). This is the angle created from the tibial plateau and the mechanical axis of the tibia, measured on the medial side.
2. The mechanical axis deviation (MAD). The width of the plateau is expressed as a percentage 0–100% medial to lateral and the point at which the mechanical axis crossed this line was measured pre- and post-operatively. The difference between this is the MAD.

### Assessment of coronal alignment of the hip

**Fig. 3:**
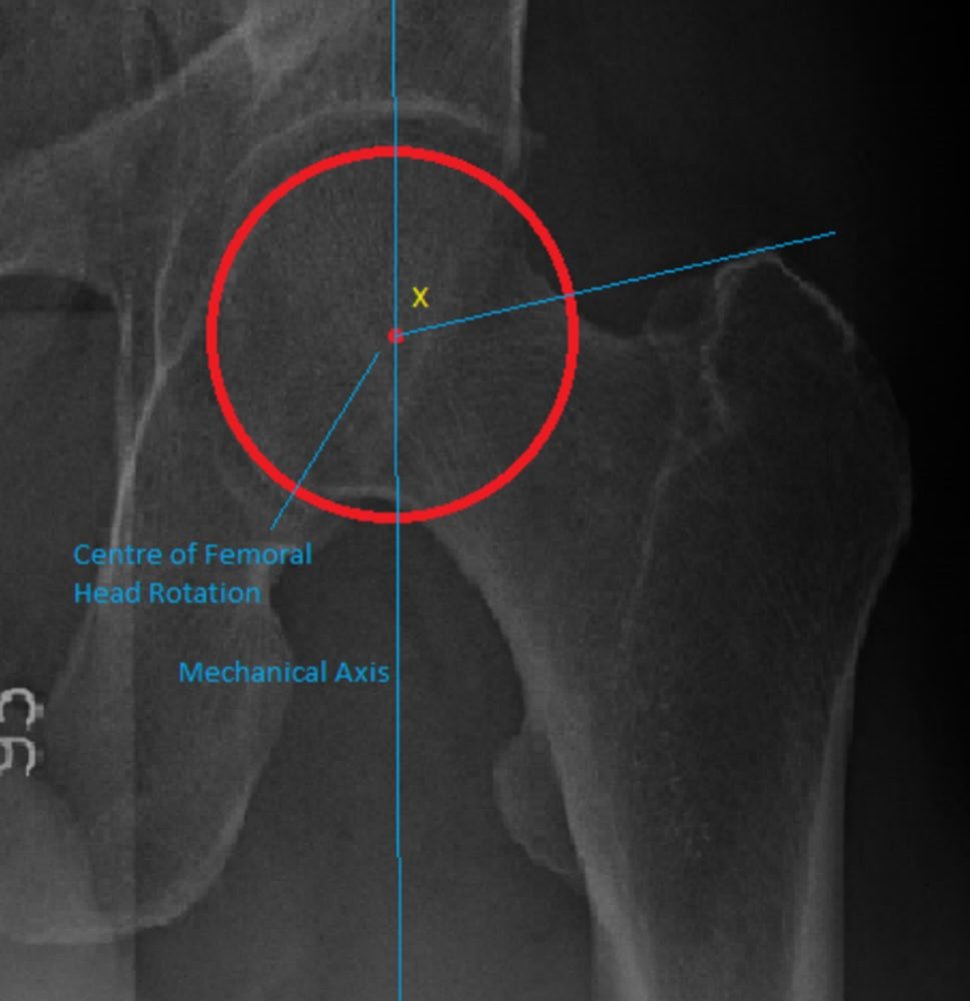
The Mechanical Greater Trochanter Angle (MGTA) is the angle created from the mechanical axis and a line from the tip of the greater trochanter to the centre of femoral head rotation (x). This angle is created by a constant line (greater trochanter to femoral head centre) to a variable line (mechanical axis). This angle was created as a novel measurement to assess the coronal changes that occur at the hip

The coronal alignment of the proximal femur itself does not change after MOHTO. For example, the neck-shaft angle is unaffected by the MOHTO. Therefore, a method to quantify changes from the osteotomy was created and is shown in Fig. 3. This is the mechanical greater trochanter angle (MGTA).

Statistical analysis was performed using SPSS for mac version 26 (SPSS Inc., Chicago, Illinois). Paired t-test or Wilcoxon signed-rank tests were performed to evaluate the change created by the osteotomy. Pearson’s correlation coefficients were calculated to determine the relationship between changes in MPTA and the MAD with MGTA. A *p*-value of < 0.05 was considered to be statistically significant.

## Results

### Patient demographics

All osteotomies were performed on male patients. The patient ages ranged from 36 to 66 *(M* = *53.79, SD* = *6.05.)* The mean height, weight and BMI were 1.76 m *(SD* = *0.63),* 96.93 kg *(SD* = *17.13)* and 31.15 *(SD* = *4.76)* respectively.

#### Assessment of corrections created by the MOHTO

The corrections created are summarised in supplementary material. The mean change in MPTA and MAD was 8.67° (SD = 3.57) and 42.19% (SD = 19.00) respectively. The MGTA changes ranged from 1.0° to 13.9° (M = 6.40°, SD 3.41). Shapiro–Wilk tests and histogram plots showed the change in MPTA, MGTA and the MAD displayed normal distribution.

#### Assessment of correlation between MPTA and MAD with MGTA

**Fig. 4:**
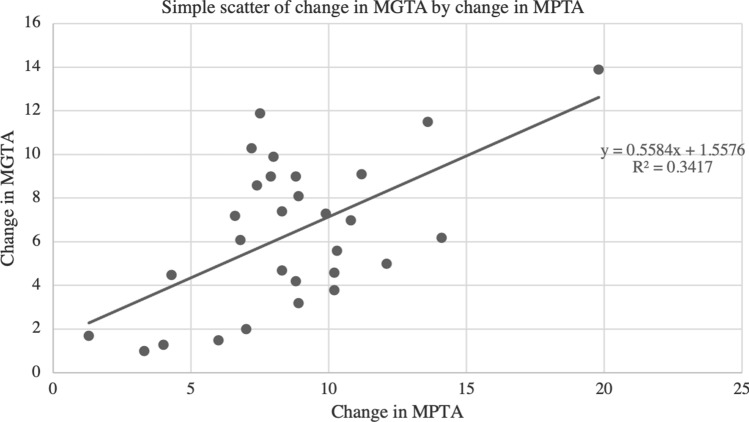
Scatter plot with best fit line comparing change in MPTA and change in MGTA. This demonstrates a moderately positive correlation (*r* = 0.585, *p* < 0.001)

**Fig. 5:**
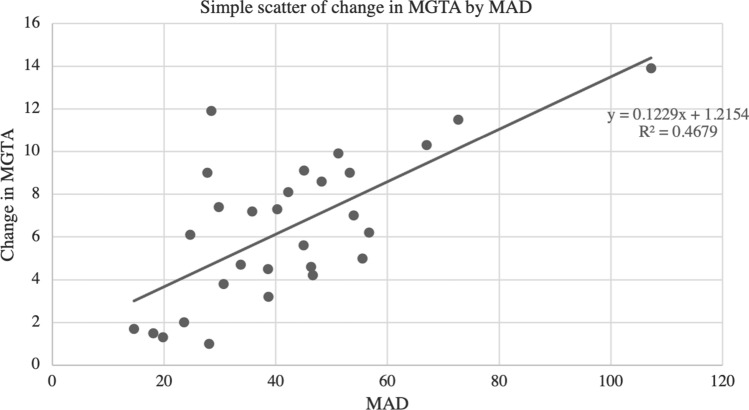
Scatter plot with best fit line comparing MAD to change in MGTA. This demonstrates a moderately positive correlation *r* = 0.684, *p* < 0.001)

Scatter plots with the line of best fit are shown in Figs. 4 and 5. A zero-order Pearson product-moment correlation test was run to determine the relationship between the change in MPTA and the change in MGTA. There was a moderately positive correlation between the change in MPTA to change in MGTA (*r* = *0.585, p* < *0.001)*. The relationship between MAD and change in MGTA was also assessed and again a zero-order Pearson product-moment revealed a moderately positive correlation between MAD and MGTA (*r* = 0.684, *p* < 0.001).

### Control for Age, weight, height, and BMI

A partial correlation was run to determine the relation whilst controlling for age, weight, height, and BMI (*r* = *0.656, p* < *0.001)* which showed these factors had little influence on the relationship between change in MPTA and change in MGTA. A partial correlation was also run to determine the relation between MAD and change in MGTA, whilst controlling for age, weight, height, and BMI (*r* = 0.762, *p* =  < 0.001), which again showed these factors had little influence on the correlation.

## Discussion

The senior author of this study observed changes in hip symptoms after MOHTO. The literature investigating changes at the hip after MOHTO is sparse. The aim of this study was to investigate whether MOHTO caused coronal alignment changes at the hip joint.

In this study, we used the change in MPTA and MAD to quantify the change created by the MOHTO. Correlation of these values with the change in MGTA showed a positive trend. That is to say the higher the correction created by the MOHTO the higher the change in the MGTA. The changes in the MGTA had a stronger correlation with the MAD than with the change in MPTA (*r* = 0.684 vs 0.585). There was very little influence from patient demographics such as age, height, weight and BMI on the observations seen. We were unable to comment on the effect of gender as all our patients were male.

This was a retrospective review of imaging and therefore the time from surgery to post-operative imaging was not standardised. This may have affected the overall results as there is some evidence to suggest loss of correction and recurrence after MOHTO. Full-length leg alignment views were not obtained for all patients that underwent a MOHTO. A total of eight patients were excluded on the basis of absent post-operative full leg length views which did reduce our sample size significantly. The sample did not include any female patients which may have affected the results of this study. A control group was not available to add as a control. For example, a closing lateral osteotomy has fallen out of favour in our institute due to the potential to cause patella height changes and leg length discrepancy. The imaging reviewed in this study did not analyse the effect of pelvic anatomy or imaging in a single leg stance. Further studies should focus on the clinical significance of these results with patient-reported outcomes and clinical assessment of hip function. Motion analysis to detect any changes in GAIT or hip function would also provide further information. We have only focused our attention on static imaging when in reality the interaction of the hip and knee is a dynamic and complex one.

The primary aim of this paper was to describe the observations found from our analysis. We are unable to comment on any clinical significance, but the findings from our study highlight the effect of MOHTO on the hip’s coronal plane alignment and the need for further research. It reminds surgeons embarking on osteotomy surgery to consider the lower limbs as a functional unit and to consider the effects of MOHTO at the hip. This is often not appreciated due to the lack of planning and the absence of literature investigating these effects. This may also be because of super specialisation, creating surgeons whose practice focuses only on a single joint, and therefore underappreciate the relevance realignment of the knee has on adjacent joints.

The changes that occur at the ankle have previously been described but this is the first study to describe the relative changes that occur at the hip [8, 9, 10, 12]. Palmer et al. have described the increased incidence of CAM deformities in patients with a failing medial knee compartment and Konrads et al. described a case of hip impingement after an HTO [5, 6]. Further studies would prove beneficial in assessing the clinical significance of these findings.

**Fig. 6:**
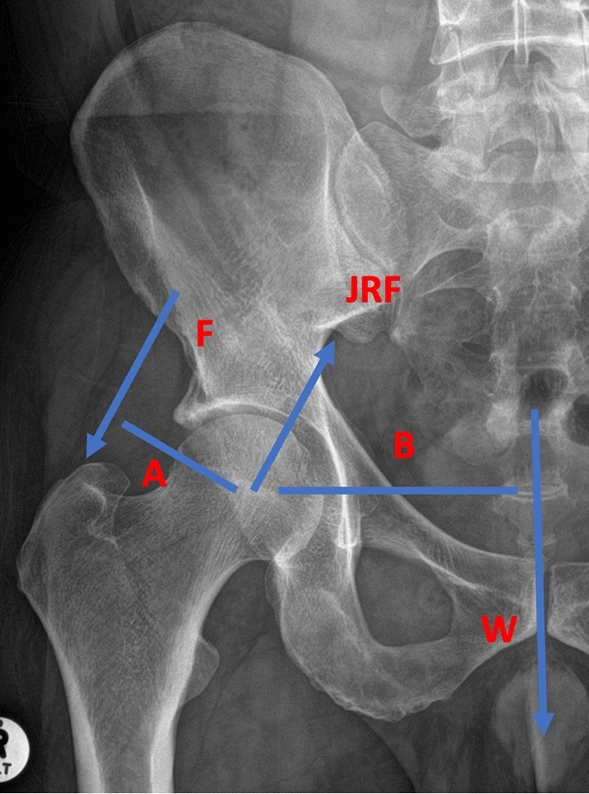
Free body diagram of the hip. F = Force of Abductor, W = Body weight, JRF = joint reaction force, A = abductor moment arm, B = moment arm of body weight. Calculating the JRF: (A x Fy) + (B x W) = 0, Ry = My + W and JRF = Ry / (cos 30°)

The biomechanical effects at the hip due to coronal plane alignment can be easily demonstrated through free body diagrams (Fig. 6). The change in the coronal alignment of the hip has the potential to change abductor muscle function. An increase in the MGTA, in theory, should increase the length and thus tension of the abductors. It will also decrease the moment arm of the abductors. It is well known from biomechanical studies of the hip that a valgus hip can increase joint reaction forces through the hip. The changes created at the hip joint demonstrated in this study may have a similar effect [13, 14]. The potential clinical significance of this is increased symptomatic hip pain from chondral damage or accelerated wear in the long run, whether this is a native or replaced hip. We can only comment on the coronal alignment changes at the hip but in reality, the effects of creating a more valgus knee is going to have an effect on other dynamic structures affecting the hip as has been described in other studies [15–17].

**Fig. 7:**
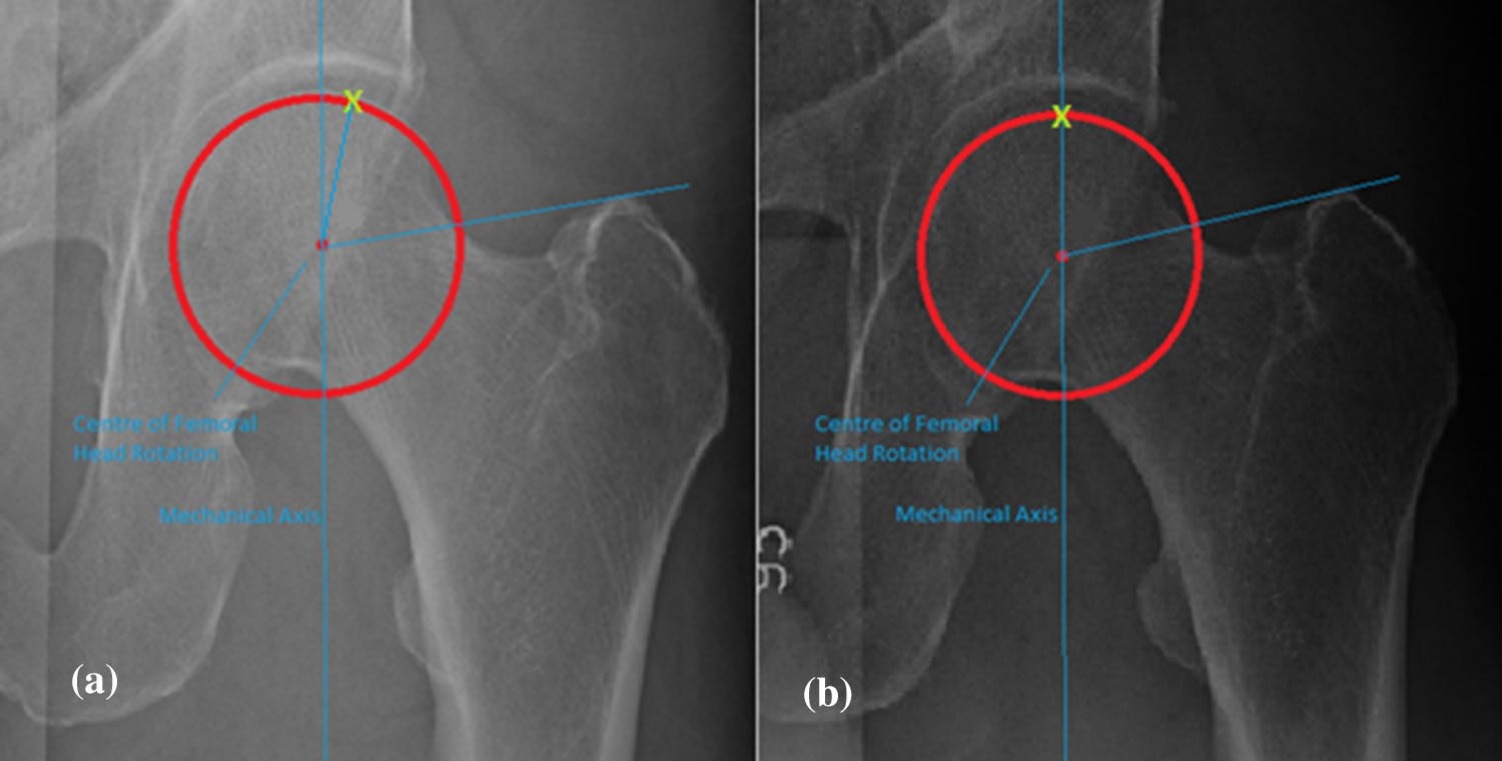
(**b**) Shows the pre op MGTA (78.8 degrees). X represents the point of the femoral head articulating at the weight bearing zone. In the postoperative image (**a**) the MGTA has increased (86.9 degrees). Point X in (**a**) shows the same area of the femoral head that was at the weight bearing zone in (**b**) but has now moved more laterally bringing a new area of the femoral head into the weight bearing zone

The weight-bearing zone of the hip changes with realignment surgery of the knee. Although previous studies have shown that the entire femoral head is involved in articulation at some point through the GAIT cycle it is common for degenerative changes to focus around the weight-bearing zone of the hip [18, 19]. The coronal alignment changes at the hip in theory will change the contact areas of the hip. For example, if the articular cartilage in the weight-bearing zone of the hip were to have underlying pathology, a change in alignment may bring normal articular cartilage into the weight-bearing zone of the hip and improve symptoms [20]. However, the converse may also be true in that a previously damaged chondral area of the hip is brought into the weight-bearing zone and becomes symptomatic. This theory is demonstrated in Fig. 7.

Impingement is a well-known cause of hip pain and can be intra- or extra-articular in causation. Previous studies have shown realignment surgery around the hip to cause a change in ischio-femoral space. The ischio-femoral space is between the ischium and lesser trochanter. Pain is often felt in the groin medially and is exacerbated by extension, adduction and external rotation of the hip. As can be seen in previous images, the ischio-femoral space is increased as a result of a valgus producing osteotomy of the knee. This has potential clinical significance in improving any previous impingement symptoms the patient may have had [21, 22].

## Conclusion

This study has highlighted that an important change in coronal alignment occurs at the hip joint after medial opening high tibial osteotomy. The changes are likely to result in an alteration in the weight-bearing portion of the femoral head and the function of the abductors. The clinical significance of these changes is not apparent thus far, but the findings described in this paper should prompt surgeons to assess the lower limb as a functional unit. Further research is needed to investigate the clinical significance of this study.

## Supplementary Information

Below is the link to the electronic supplementary material.

Supplementary file1 (XLSX 12 kb)
